# Nontoxigenic Highly Pathogenic Clone of *Corynebacterium diphtheriae*, Poland, 2004–2012

**DOI:** 10.3201/eid1911.130297

**Published:** 2013-11

**Authors:** Aleksandra A. Zasada

**Affiliations:** National Institute of Public Health–National Institute of Hygiene, Warsaw, Poland

## Abstract

Twenty-five cases of nontoxigenic *Corynebacterium diphtheriae* infection were recorded in Poland during 2004–2012, of which 18 were invasive. Alcoholism, homelessness, hepatic cirrhosis, and dental caries were predisposing factors for infection. However, for 17% of cases, no concomitant diseases or predisposing factors were found.

*Corynebacterium diphtheriae* is the causative agent of diphtheria. Its toxin is considered the major virulence factor. Since introduction of vaccine against the diphtheria toxin in the 1940s, infections caused by toxigenic corynebacteria have been well controlled in industrialized countries that have high coverage rates of childhood vaccination with 3 doses of diphtheria-tetanus-pertussis vaccine (*1*). Nevertheless, emergence of nontoxigenic *C. diphtheriae* infections has been reported in some of these countries.

In line with other European countries, Poland routinely vaccinates against diphtheria (Technical Appendix). According to data from the World Health Organization, >95% of children in Poland are fully vaccinated against diphtheria. The last diphtheria case was recorded there in 2000 (http://www.who.int/immunization_monitoring/data/incidence_series.xls).

The absence of diphtheria during the past 13 years suggests that the high vaccination coverage rates in Poland protect against diphtheria. In 2004, the first case of sepsis caused by nontoxigenic *C. diphtheriae* was recorded (*2*). Other cases were recorded in 2006, and since 2007, several cases of *C. diphtheriae* invasive infections have been recorded every year (Table 1). In addition, local infections—usually wound infections—caused by nontoxigenic *C. diphtheriae* were recorded (Table 2). A total of 25 nontoxigenic *C. diphtheriae* infections were recorded in Poland in 2004–2012, of which 18 were invasive infections.

**Table 1:** Cases of bloodstream infections caused by nontoxigenic *Corynebacterium diphtheriae*, Poland, 2004–2012

Patient	Age, y/sex	Concomitant disease	Location	Year	Additional information
Inv-01	38/M	Dental caries	Warsaw	2004	Endocarditis diagnosed
Inv-02	ND/M	ND	Bydgoszcz	2006	Homeless
Inv-03	51/M	HIV suspected	Gdynia	2007	
Inv-04	37/M	Dental caries	Gdynia	2007	Endocarditis diagnosed
Inv-05	53/M	Alcoholism, hepatic cirrhosis	Rzeszów	2008	
Inv-06	50/F	Portal and posthepatitic C cirrhosis, dental caries	Bydgoszcz	2008	
Inv-07	32/M	Alcoholism, abscess of the liver	Gdynia	2008	
Inv-08	24/M	Not identified	Gdynia	2009	
Inv-09	17/M	Not identified	Kraków	2009	
Inv-10	60/M	Alcoholism	Sosnowiec	2009	
Inv-11	60/M	Dental caries, frostbite of feet	Bydgoszcz	2010	Homeless
Inv-12	36/M	Alcoholism, delirium	Gdynia	2010	
Inv-13	37/M	Not identified	Legnica	2010	Endocarditis diagnosed
Inv-14	ND/M	ND	Sosnowiec	2011	
Inv-15	50/M	Skull trauma, skin ulceration	Radom	2011	Homeless
Inv-16	71/M	Stroke	Kraków	2011	
Inv-17	65/M	Hepatic cirrhosis, encephalopathy, diabetes mellitus	Gdańsk	2012	
Inv-18	ND/M	Stroke	Poznań	2012	Endocarditis diagnosed

ND, no data.

**Table 2:** Local infections caused by nontoxigenic *Corynebacterium diphtheriae*, Poland, 2004–2012

Patient	Age, y/sex	Location	Year	Site of *C. diphtheriae* isolation
Loc-01	ND/M	Bydgoszcz	2007	Wound
Loc-02	29/F	Warszawa	2007	Fistula
Loc-03	ND/M	Bydgoszcz	2007	Wound
Loc-04	51/M	Warszawa	2008	Wound
Loc-05	61/F	Bydgoszcz	2010	Shank cyst
Loc-06	56/F	Gdynia	2010	Wound
Loc-07	59/M	Warszawa	2012	Wound

ND, no data.

## The Study

All patients were admitted to local hospitals and clinical samples for microbiological investigations were sent to the nearest laboratories. *C. diphtheriae* isolates were sent to National Institute of Public Health–National Institute of Hygiene for confirmation and toxigenicity testing, biotyping, pulsed-field gel electrophoresis (PFGE), multilocus sequence typing (MLST), and ribotyping. Case classification and microbiological methods used are presented in the Technical Appendix. Data collected for epidemiologic analysis included location; type of infection; year of presentation; and patient age, sex, concomitant diseases, socioeconomic status, and intravenous drug use (IVDU).

All isolates from local and invasive infections were identified as biotype *gravis*, except for the isolate from patient Loc-05, which was identified as biotype *mitis*. All 25 isolates were characterized by PFGE, and 20 isolates (18 from invasive and 2 from local infections) were characterized by MLST (*3*–*5*). All the isolates except the *mitis* isolate belonged to the same pulsotype revealed by PFGE. All the isolates characterized by MLST belonged to genotype sequence type 8. Eight of the isolates (5 from invasive and 3 from local infections) also were genotyped by using ribotyping. All 8 isolates showed indistinguishable ribotype patterns (*3*).

All but 1 invasive infection were identified in male patients, whereas local infections affected male and female patients similarly. Age groups of patients most affected by invasive infections were 31–40 years, followed by 51–60 years; for local infections, persons 51–60 years of age were mostly affected (Figure). Patients’ ages ranged from 17 to 71 years. The cases occurred in various parts of Poland; no epidemiologic links were identified.

**Figure:**
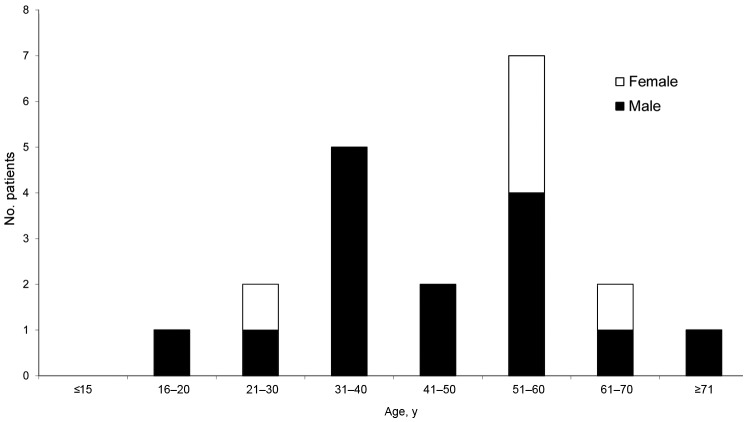
Number of nontoxigenic *Corynebacterium diphtheriae* infections, Poland, 2004–2012. Excluded are 5 cases for which no data were available.

Epidemiologic data analysis revealed that predisposing factors of nontoxigenic *C. diphtheriae* invasive infections were related to conditions associated with low socioeconomic status, such as alcoholism, homelessness, and dental caries, as well as to hepatic cirrhosis. For 3 (17%) patients (Inv-08, Inv-09, Inv-13), no concomitant diseases or predisposing factors were identified. These were healthy men aged 17, 24, and 37 years of age, respectively. Predisposing factors for local infections were not analyzed. The sources of all infections described in the study were not identified. Despite IVDU being regarded as a risk factor for *C. diphtheriae* invasive infection, none of the patients were intravenous drug users.

## Conclusions

Diphtheria is a rare disease in Europe. In 2006–2009 only 150 cases were reported in European Union and European Economic Area/European Free Trade Association countries. Most of the cases (114 cases) occurred in Latvia, where diphtheria is endemic. The other diphtheria cases were reported in France (10 cases), Germany (6), Sweden (2), United Kingdom (16), and Norway (4) (*6*). However, infections from nontoxigenic *C. diphtheriae* have been reported in several European countries, such as Germany (*7*), United Kingdom (*8*), France (*5*), Switzerland (*9*), and Italy (*10*), during the past few years. In Poland, persons most affected were 31–40 years and 51–60 years of age, whereas in other countries most patients were younger (up to 34 years of age). No *C. diphtheriae* infections among children were recorded in Poland, whereas in France, almost 20% of invasive infections were diagnosed in children. On the other hand, in Italy and the United Kingdom, 70% and 13% of isolates, respectively, originated from patients <15 years of age (*5*,*8*,*10*).

In Poland, all but 1 strain isolated from local and invasive infections belonged to biotype *gravis*, whereas biotype *mitis* dominated among the invasive isolates in Switzerland and France, and biotype *gravis* dominated among isolates from local infections in Italy and the United Kingdom (*5*,*8*–*10*). All but 1 isolate from Poland represent a single clone despite isolation of the strains in different part of the country over a 9-year period. This phenomenon has not been documented in any countries reporting nontoxigenic *C. diphtheriae* infections. This raises a valid question: is a single clone of *C. diphtheriae* circulating in Poland or does the identified clone have increased pathogenic properties? This question remains unanswered because the carrier state of *C. diphtheriae* has not been examined in the Polish population.

Taking these data and the literature review into consideration, *C. diphtheriae* infections frequently are associated with endocarditis. Muttaiyah et al. (*11*) and Mishra et al. (*12*) demonstrated that most patients with *C. diphtheriae* endocarditis have underlying cardiac disease, prosthetic valves, or a history of IVDU. This finding, however, was not observed among patients in Poland.

The portal of entry for invasive nontoxigenic *C. diphtheriae* infection has not been fully elucidated. However, some authors shown that skin lesions are the most likely sources (*9*,*13*,*14*). In the cases presented here, skin ulceration was uncommon (1 case), but dental caries were found in >22% of cases. Dental caries could be a portal of entry.

The main limitation of this work is lack of complete data. Nevertheless, nontoxigenic *C. diphtheriae* can be concluded to be an emerging pathogen in Poland and has the potential to cause serious infections. The number of nontoxigenic *C. diphtheriae* infections might be higher because reporting of only toxigenic *C. diphtheriae* infections is mandatory in Poland. Moreover, in clinical settings, detection of coryneform bacteria in blood cultures is often dismissed as contamination, and in severe cases of the disease, *C. diphtheriae* might never be identified as the etiologic agent of bloodstream infection.

Homelessness, alcohol abuse, IVDU, and diabetes mellitus were mentioned in the literature as risk factors for *C. diphtheriae* invasive infections. In the cases presented here, 31% of patients were homeless, and 22% reported alcohol dependency but only 1 patient had diabetes mellitus. No patients reported IVDU. In 17% of cases, hepatic cirrhosis was ascertained, which suggests that it also may be another predisposing factor to infection. Moreover, dental caries is a highly probable portal of entry of *C. diphtheriae* invasive infection and has not been documented by other authors. However, such infections also might occur in persons with no identified predisposing factors.

Technical Appendix Diphtheria toxoid vaccination schedule in Poland, case classification, and methods of identification and characterization of the isolates.
