# Supplementary material for: Nontoxigenic Highly Pathogenic Clone of Corynebacterium diphtheriae, Poland, 2004–2012
Source: Emerg Infect Dis. 2013 Nov;19(11):1870–2. doi: 10.3201/eid1911.130297 (PMC3837660; doi:10.3201/eid1911.130297)
Supplement: Technical Appendix — Diphtheria toxoid vaccination schedule in Poland, case classification, and methods of identification and characterization of the isolates. [file 13-0297-Techapp-s1.pdf]

Article DOI: http://dx.doi.org/10.3201/eid1911.130297

Nontoxigenic Highly Pathogenic Clone of *Corynebacterium diphtheriae*, Poland, 2004–2012

Technical Appendix

Diphtheria Toxoid Vaccination Schedule in Poland

Diphtheria vaccination schedule comprises 7 doses administered at ages 2 months; 3–4 months; 5–6 months; 16–18 months; and then 6, 14, and 19 years. In adults, booster doses every 10 years are recommended.

Case Classification

Nontoxigenic *C. diphtheriae* invasive infection was diagnosed when nontoxigenic *C. diphtheriae* were isolated from 3 separately taken blood samples.

Nontoxigenic *C. diphtheriae* local infection was diagnosed when nontoxigenic *C. diphtheriae* were isolated from the sample (e.g., wound swabs) as the only or dominating microorganism.

Methods of Identification and Characterization of the Isolates

The isolates were identified by Gram staining, colonial morphology, and biochemical ApiCoryne tests (bioMerieux, Marcy l'Etoile, France). Toxin production was investigated by using conventional and modified Elek test as described (*1*). Genotyping was performed as described for ribotyping (*2*), pulsed-field gel electrophoresis (*3*), and multilocus sequence typing (*4*).

References

1. Zasada AA, Zaleska M, Podlasin RB, Seferyńska I. The first case of septicemia and endocarditis due to nontoxigenic *Corynebacterium diphtheriae* in Poland. Ann Clin Microbiol Antimicrob. 2005;4:8. PubMed http://dx.doi.org/10.1186/1476-0711-4-8

2. Zasada AA, Baczewska-Rej M, Wardak S. An increase in non-toxigenic *Corynebacterium diphtheriae* infections in Poland—molecular epidemiology and antimicrobial susceptibility of

strains isolated from past outbreaks and those currently circulating in Poland. Int J Infect Dis. 2010;14:e907–12. PubMed http://dx.doi.org/10.1016/j.ijid.2010.05.013

3. Zasada AA, Baczewska-Rej M. Types of *Corynebacterium diphtheriae* strains isolated in Poland in 2004–2008 [in Polish]. Med Dosw Mikrobiol. 2008;60:183–90. PubMed

4. Farfour E, Badell E, Zasada A, Hotzel H, Tomaso H, Guillot S, et al. Characterization and comparison of invasive *Corynebacterium diphtheriae* isolates from France and Poland. J Clin Microbiol. 2012;50:173–5. PubMed http://dx.doi.org/10.1128/JCM.05811-11
